# Calcium-activated SK channels control firing regularity by modulating sodium channel availability in midbrain dopamine neurons

**DOI:** 10.1038/s41598-017-05578-5

**Published:** 2017-07-12

**Authors:** Rajeshwari Iyer, Mark A. Ungless, Aldo A. Faisal

**Affiliations:** 1 MRC London Institute of Medical Sciences (LMS), Hammersmith Hospital Campus, Du Cane Road, London, W12 0NN UK; 2 Institute of Clinical Sciences (ICS), Faculty of Medicine, Imperial College London, Du Cane Road, London, W12 0NN UK; 3 Department of Bioengineering, Imperial College London, London, United Kingdom; 4 Department of Computing, Imperial College London, London, United Kingdom

## Abstract

Dopamine neurons in the substantia nigra pars compacta and ventral tegmental area regulate behaviours such as reward-related learning, and motor control. Dysfunction of these neurons is implicated in Schizophrenia, addiction to drugs, and Parkinson’s disease. While some dopamine neurons fire single spikes at regular intervals, others fire irregular single spikes interspersed with bursts. Pharmacological inhibition of calcium-activated potassium (SK) channels increases the variability in their firing pattern, sometimes also increasing the number of spikes fired in bursts, indicating that SK channels play an important role in maintaining dopamine neuron firing regularity and burst firing. However, the exact mechanisms underlying these effects are still unclear. Here, we develop a biophysical model of a dopamine neuron incorporating ion channel stochasticity that enabled the analysis of availability of ion channels in multiple states during spiking. We find that decreased firing regularity is primarily due to a significant decrease in the AHP that in turn resulted in a reduction in the fraction of available voltage-gated sodium channels due to insufficient recovery from inactivation. Our model further predicts that inhibition of SK channels results in a depolarisation of action potential threshold along with an increase in its variability.

## Introduction

Dopamine neurons from the midbrain, especially from the substantia nigra pars compacta (SNc) and the ventral tegmental area (VTA) have a significant influence on target neurons due to their extensive axonal arbours^1^ projecting to a large number of regions in the brain^2^ and therefore regulate important day-to-day functions like fine motor control^3, 4^, learning and memory^5–7^, reward-related and motivational behaviour^7–9^, and response to stress and noxious stimuli^10–12^. Dysfunction in these neurons has been linked to several disorders such as Schizophrenia^13–15^, rewarding effects of alcohol and drugs of abuse^6, 16, 17^, and Parkinson’s Disease (PD) that results in a loss of fine motor control and other cognitive deficits^18^.

Dopamine neurons display two principal types of spontaneous firing patterns *in vivo* – single spike firing and burst firing. Single spike firing is characterised by single action potentials fired at irregular (or regular) intervals while burst firing is composed of two to ten spikes fired in close succession interspersed with irregular single spikes^19, 20^. Although midbrain dopamine neurons display heterogeneity in their firing properties^21^, single spike firing, with varying degrees of regularity in inter spike intervals (ISI), is the more commonly observed firing pattern in dopamine neurons, especially in the SNc compared to the VTA, and has been hypothesised to help maintain ‘tonic’ dopamine levels in target regions like the striatum^6, 22^. The regularity of single spike firing, both *in vivo* and *in vitro*, appears to be controlled by the small conductance calcium activated potassium (SK) channels, because when these channels are inhibited by apamin, firing regularity is decreased as quantified by an increase in the coefficient of variation (CV) of ISI^23, 24^. This effect appears to be a result of a reduction in the post-spike after-hyperpolarisation (AHP) mediated by SK currents. Although the physiological importance of maintaining firing regularity in dopamine neurons has not specifically been explored in behavioural experiments, decreased firing regularity in cerebellar Purkinje neurons, as a result of insufficient SK channel function, has been linked to decreased motor co-ordination in an ataxia mouse model^25^. Moreover, decreased firing regularity associated with increased burst firing due to reduced SK channel function has also been reported in SNc dopamine neurons in mice models of PD^26^. The potassium current that flows through SK channels hyperpolarises the neuronal membrane after each spike thus enabling other voltage-gated ion channels such as sodium to recover from inactivation and increase the number of available channels for activation^27^ when the next wave of depolarisation occurs resulting in the next spike. When SK channels are inhibited, it is intuitive that the firing rate would increase due to the reduction in post-spike AHP, however, why should this result in a decrease in firing regularity?

A computational modelling study recently demonstrated a reduced availability of sub-threshold activated sodium and potassium channels in the presence of SK channel blockade due to reduced AHP, resulting in a decrease in firing regularity in globus pallidus neurons^28^. While a decrease in firing regularity due to SK channel inhibition has been replicated in many experiments, a hypothesis for what the underlying mechanism mediating this effect could be has not yet been proposed for midbrain dopamine neurons. Therefore we hypothesised that, due to the reduction in post-spike AHP after SK channel inhibition, sometimes enough sodium channels recover from inactivation and in turn the neuron fires a spike but sometimes majority may still remain inactivated and therefore no spikes will be fired, thus resulting in an irregular spike train.

To test this hypothesis, we built a computational dopamine neuron model where ion channel gating was modelled as a *stochastic* continuous-time Markov chain^29, 30^ in order to replicate the physiologically realistic probabilistic transitions between the different conformations ion channels are capable of existing in, such as, *open*, *inactivated*, and *closed*. This is different from existing *deterministic* models of midbrain dopamine neurons that model ion channels as single lumped conductances that do not take into account the random transitions between states^31–40^ and therefore do not display variability in firing. Our model is also different from^28^ who introduced stochasticity using a noisy leak conductance. Moreover due to the relatively small number of channels found in midbrain dopamine neurons^41^, we expected that adding channel stochasticity would enable us to demonstrate how a reduction in post-spike AHP further reduces the availability of spike-generating ion channels, thereby resulting in a reduction in firing regularity. For example, the small number of *open* ion channels (as opposed to the total number of ion channels) is critical when it comes to firing action potentials in very small diameter patches of neuron where even a single open sodium channel can give rise to spontaneous or ‘random’ action potentials if it stays open for long enough, which in fact places a lower limit to how small an axon can be ref. 42. Ion channel stochasticity has also been shown to account for changes in action potential shape that depends, to a large extent, on the number of available or *open* sodium and potassium channels during the course of the action potential^42, 43^. The variability in action potential threshold is also in part due to the small number of *open* ion channels around action potential threshold that determines the reliability in action potential initiation and precision^44–46^, reinforcing the idea that ion channel noise is important for firing regularity.

Indeed, using our novel computational model of a dopamine neuron incorporating ion channel stochasticity, we show that with SK channel inhibition, reduced post-spike AHP results in an overall reduction of number of *open* sodium channels as a result of a greater and variable number of these channels remaining in the inactivated state, thereby resulting in an irregular spike train. We further demonstrate that such a reduced recovery from inactivation of sodium channels also results in the overall depolarisation of spike threshold. The reduced AHP also results in a reduction in the A-type potassium current that could in part explain the increased rate of firing that often accompanies SK-channel inhibition.

## Results

### Spontaneous spike train characteristics *in silico*

**Figure 1:**
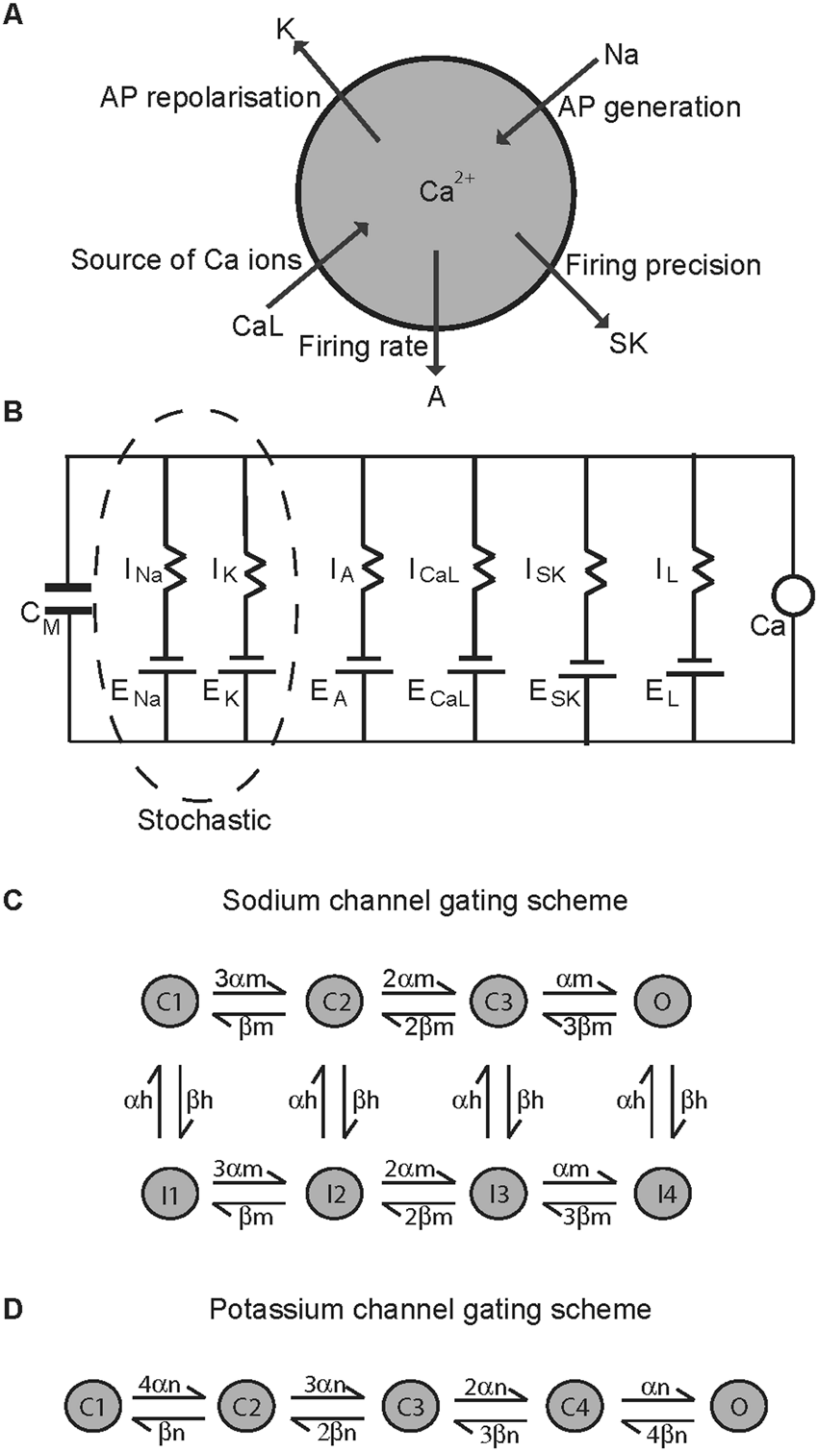
Dopamine neuron model. (**A**) A single spherical compartment of diameter 10 μm and capacitance 1 μF represents the dopamine neuron soma. It is incorporated with those ionic conductances known to be sufficient to reproduce the basic electrophysiological features associated with these neurons, such as the Na (sodium channel) and K (delayed rectifier potassium channel) that regulate the generation and repolarisation of the action potential respectively; the A (A-type potassium channel) that helps maintain firing frequency; CaL (L-type calcium channel) that provides the bulk of intracellular calcium; SK (calcium-activated potassium channel of the SK type) that maintains firing regularity. (**B**) The Hodgkin-Huxley style equivalent electrical circuit for the model schematic in (**A**). The sodium and delayed rectifier potassium channels are modelled as stochastic, keeping all other ion channels deterministic. The neuronal membrane is considered as a capacitor with capacitance C_M_ and with ion channels in parallel to each other. E_ion_ is the electromotive force that represents the reversal potential of the respective ion channel, I_ion_ represents the current flowing through them, and Ca is the intracellular calcium ion concentration. (**C**,**D**) Markov gating schemes for sodium and potassium channels respectively. Each sodium channel can exist in one of activated/open, inactivated or closed states while each potassium channel can either be open or closed.

**Figure 2:**
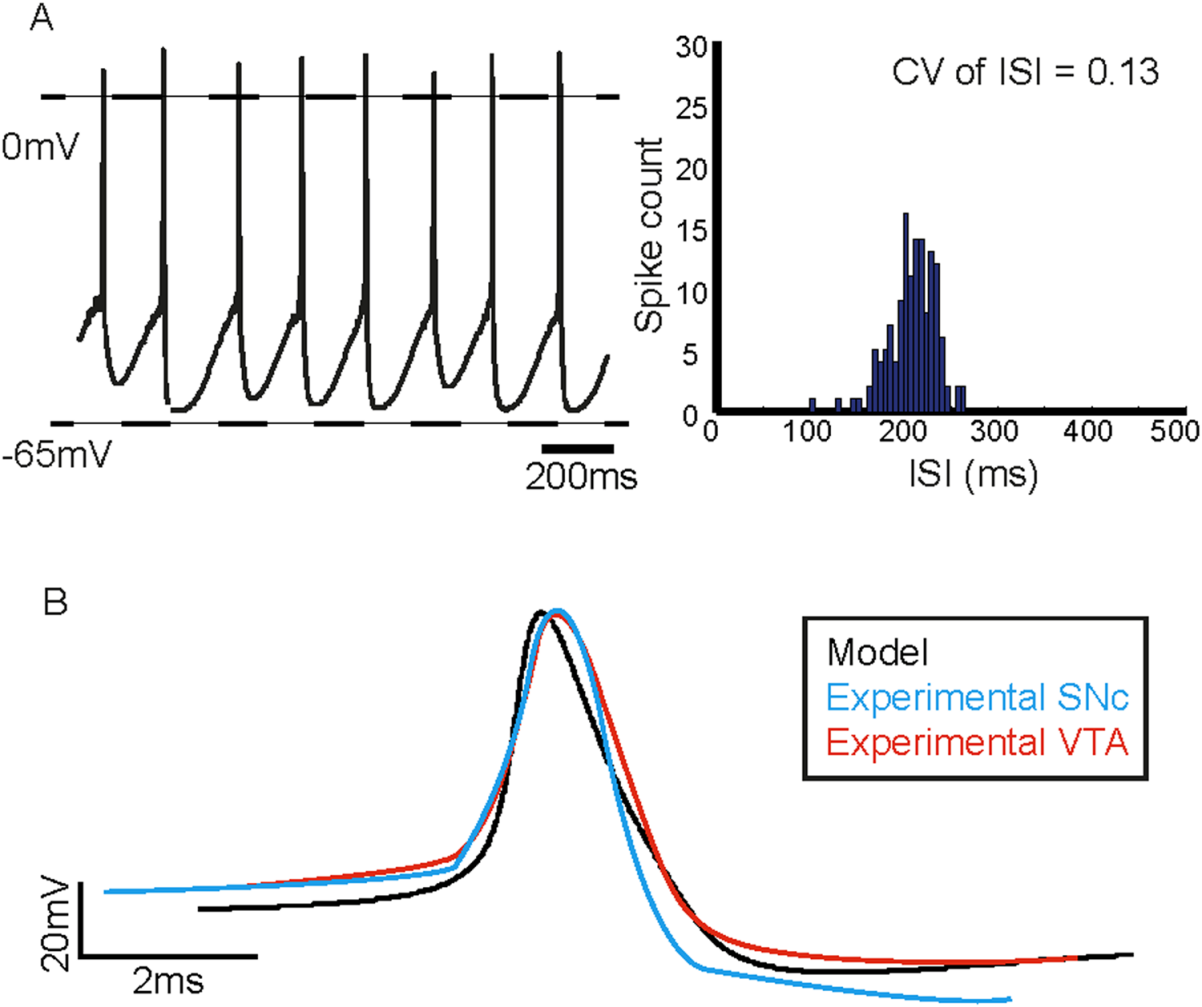
Spontaneous spike train and action potential characteristics obtained from simulations. (**A**) The *in silico* dopamine neuron fired spontaneously at a rate of 3.6 Hz and the CV of ISIs is equal to 0.13. The spike train was characterised by slow depolarisation preceding the spike, followed by a pronounced post-spike AHP. (**B**) Average action potential waveforms obtained from the model superimposed with those obtained from the SNc and VTA. The average waveforms were aligned at the point of maximum rate of rise. (Experimental waveforms adapted from ref. 55).

Our model dopamine neuron (Fig. 1) fired at a slow spontaneous rate of 3.6 Hz (Fig. 2A (*left*)), that closely resembles some of the average values reported experimentally (3.1 ± 0.9 Hz^47^, 3.7 ± 0.67 Hz^10^, 3.43 ± 0.25 Hz^48^, 4.5 ± 1.7 Hz^20^ and within the range 1–8 Hz^49^. The CV of ISI was 0.13 (Fig. 2A (*right*)), which also falls within ranges reported in experiments for dopamine neurons showing a ‘regular’ firing pattern for example, 0.01–0.02^50^, 0.06 and 0.18^51^, 0.13^52^. Action potentials were broad with a half-maximal width of 2.4 ms (2.7 ± 0.5 ms^53^; 1.74 ± 0.7 ms to 2.71 ± 0.19 ms^54^ and were characterised by a slowly rising depolarisation preceding the action potential followed by a prolonged AHP (Fig. 2B). An average action potential obtained from the simulations is compared to those obtained from experiments in Fig. 2B.

Slow firing rate and broad action potentials are considered hallmark electrophysiological properties that are commonly used to identify putative dopamine neurons in the midbrain^53, 55–58^ and since our model reproduced these features, we believe that the model captures ion channel dynamics underlying these features of firing. We therefore next wanted to test if the model dopamine neuron responds to simulated pharmacological manipulations in a manner similar to those seen in experiments in order to further demonstrate that the model is a good representation of an average midbrain dopamine neuron.

### Response to simulated pharmacological manipulations

#### The A-type potassium channel controls spontaneous firing frequency

**Figure 3:**
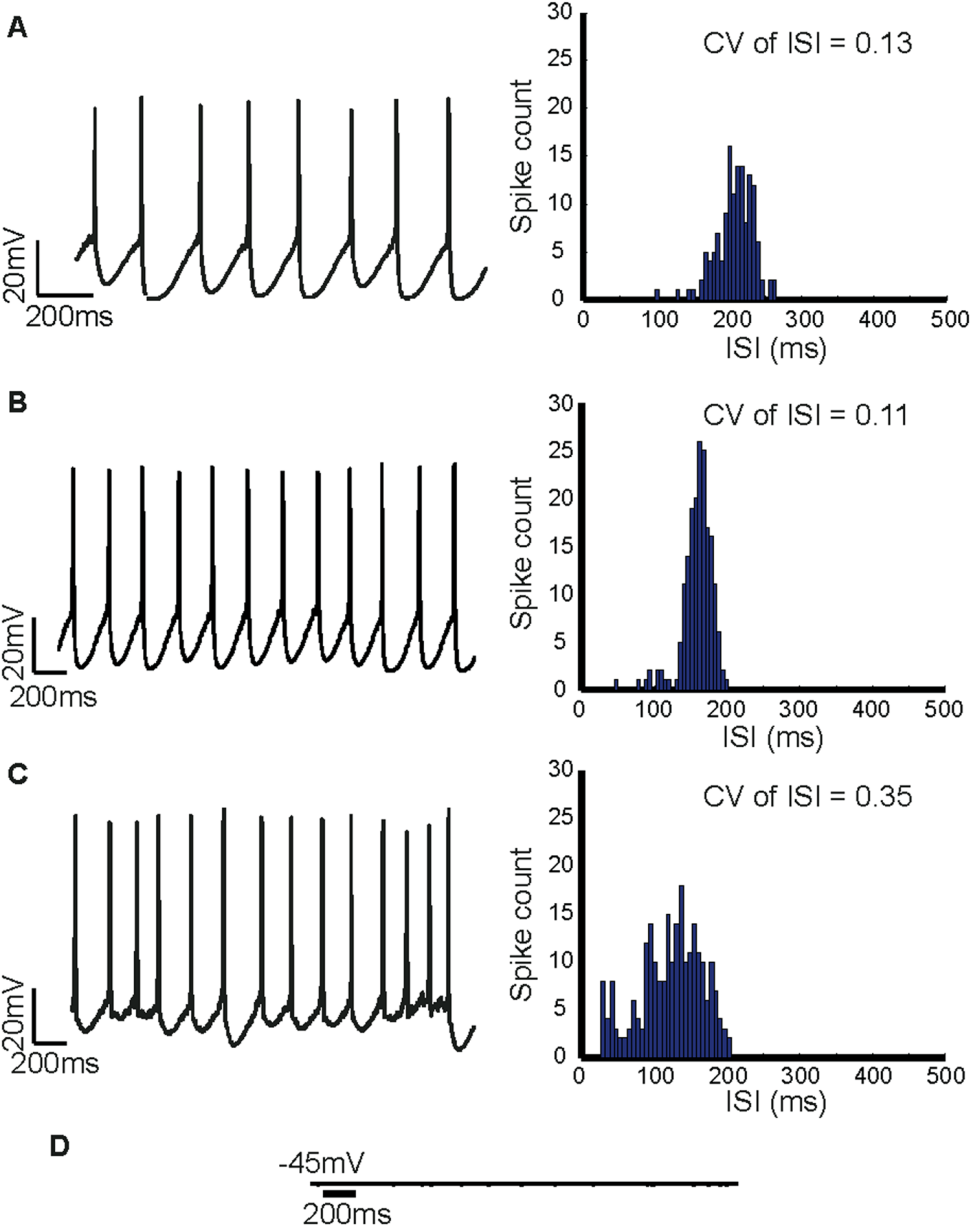
Effects of simulated inhibition of ion channels regulating spontaneous firing in the model dopamine neuron. (**A**) Under control conditions the *in silico* neuron fired spontaneously at a rate of 3.6 Hz (*left*). (**B**) When the maximal conductance of A-type potassium channels is reduced by 50% from 4 mS/cm^2^ to 2 mS/cm^2^ we observed a 1.8% increase in firing rate. As we can see from the ISI histogram, there was no change in the CV of ISI, indicating that A-type channels are involved in regulating firing rate and not firing regularity. (**C**) When the L-type calcium channel was inhibited by reducing the maximal conductance from 15 mS/cm^2^ to 5 mS/cm^2^ there was a marked decrease in firing regularity as can be seen from the spike train (*left*) and the ISI histogram. The CV of ISI increased from 0.13 to 0.35, which is a similar increase observed during SK channel inhibition. (**D**) Simulating a sodium channel block resulted in a cessation of firing indicating that sodium channels are essential for spike production.

A-type potassium channels have been shown to regulate firing frequency by opposing the depolarising current during an ISI in order to delay the subsequent action potential^59^. They are also involved in regulating action potential duration^60^ and latency to first action potential after recovery from membrane hyperpolarisation^61, 62^. Midbrain dopamine neurons express A-type transient potassium currents^61, 63, 64^ and have been associated with regulation of pacemaker firing frequency^64, 65^. Simulating an inhibition of A-type potassium channels in the model neuron by reducing its maximal conductance by 50% from 4 mS/cm^2^ to 2 mS/cm^2^ increased the firing frequency by 1.8-fold from 3.6 Hz (Fig. 3A (*left*)) in control to 5.4 Hz (Fig. 3B (*left*)). While this is similar to some of the reported values^66^ it is in contrast to a nearly three-fold increase observed in ref. 64. The increase in firing rate did not affect the firing regularity as seen in Fig. 3B ^67^, compared to control (Fig. 3A (*right*)).

#### Inhibiting L-type calcium channels decreases firing regularity

Midbrain dopamine neurons especially in the SNc fire single spikes in a regular manner. One of the mechanisms underlying this regular firing is a sub-threshold oscillatory potential, depolarised by L-type calcium channel current and repolarised by SK channel current^31, 35^ and blocking L-type calcium channels results in the abolishment of spontaneous firing^68, 69^. However, some studies have also shown that such a pacemaker-like firing can continue even in the absence of L-type calcium channel current with co-operation from other ion channel types^35, 36^ suggesting that L-type channels are not essential for spontaneous firing.

In our model, inhibition of the L-type calcium channel in the model neuron resulted in a decrease in firing regularity (Fig. 3C (*left*)), similar to the decrease in firing regularity observed with inhibition of SK channels. This is because L-type calcium channels are the only source of intracellular calcium used to activate SK channels in our model neuron. Reducing the maximal conductance of the L-type calcium channel from 15 mS/cm^2^ to 5 mS/cm^2^ results in an increase in CV of ISI to 0.35 (Fig. 3C (*right*)) from 0.13 in control (Fig. 3A (*right*)). Such a reduction in firing regularity with L-type calcium channel block is not seen in experiments^70^, perhaps due to the presence of other calcium channels.

#### Sodium channel inhibition abolishes spontaneous firing

Sodium channels are essential for spike production in midbrain dopamine neurons^41, 71^. In agreement with this, our *in silico* neuron stopped firing and the membrane potential rested at −45 mV when the sodium channel conductance was reduced to zero (Fig. 3D) simulating a sodium channel block due to TTX (tetrodoxin; a selective sodium channel blocker). While some dopamine neurons especially in the SNc continue to oscillate at a sub-threshold level after sodium channel inhibition in TTX^41, 53, 72, 73^, not all dopamine neurons exhibit this oscillatory behaviour, especially in the VTA^74, 75^.

### Simulated inhibition of SK channels decreases firing regularity and increases firing rate

**Figure 4:**
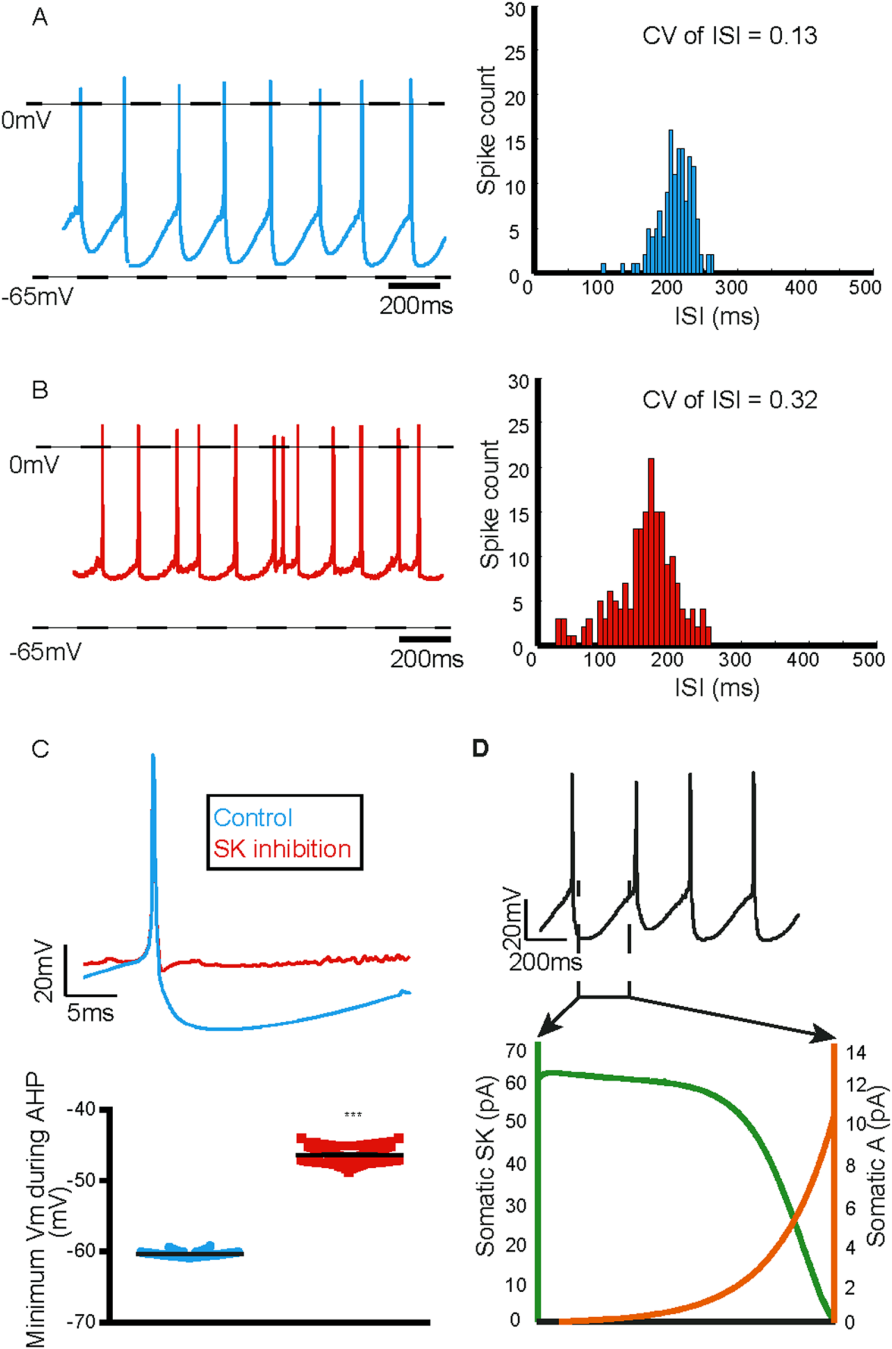
Simulated inhibition of SK channels decreases firing regularity and increases firing rate. (**A**) The model dopamine neuron fired spontaneously at a slow and rhythmic rate of 3.6 Hz (*left*) with CV of ISI = 0.13 (*right*). (**B**) Simulated SK channel inhibition resulted in a decrease in the firing regularity (*left*) and the CV of ISI increased to 0.32 (*right*). (**C**) This was accompanied by a decrease in the AHP of the action potential (*top*), quantified by a significant decrease in peak AHP (*bottom*). (**D**) Somatic currents prominent during the ISI. Error bars represent mean ± S.E.M, not visible due to small errors.

While the model produced a spontaneous regular spike train (Fig. 4A (*left*)), a simulated inhibition of the SK channel (reducing SK conductance from 5 mS/cm^2^ to 0.5 mS/cm^2^) resulted in a significant decrease in firing regularity (Fig. 4B (*left*)). Decrease in firing regularity is apparent in the spike train as well as quantified by an increase in the CV of ISI to 0.32 (Fig. 4B (*right*)) from 0.13 (Fig. 4A (*right*)). This decrease in regularity is accompanied by a significant decrease in the amplitude of the AHP following the spikes (peak AHP: without SK channel inhibition = −60.31 ± 0.03 mV; with SK channel inhibition = −46.36 ± 0.06 mV, p < 0.05) (Fig. 4C) as compared to control. This result indicated that the AHP provided by the SK current is likely to be crucial for maintaining the firing regularity in these neurons, as has been demonstrated in experiments.

We also observed an increase in the firing rate after a simulated block of SK channels. The firing rate increased by ~36% from 3.6 Hz to 5.6 Hz (Fig. 4A,B (*left*)). This is in line with increase in firing rate seen with a pharmacological block of SK channels using apamin in several experiments^52, 76, 77^. However sometimes either a decrease or no significant change in firing rate is observed^47, 78^. The discrepancy in firing rate changes observed experimentally could be attributed to a differential expression of SK2 (both firing rate and regularity) and SK3 (firing rate alone) subtypes as demonstrated in a gene-knockout study^77^. It is intuitive that SK channel inhibition would result in an increase in firing rate, as it is the most prominent current during the ISI (Fig. 4D) that prevents depolarisation to next action potential.

### SK channels determine action potential threshold and minimise its variability

**Figure 5:**
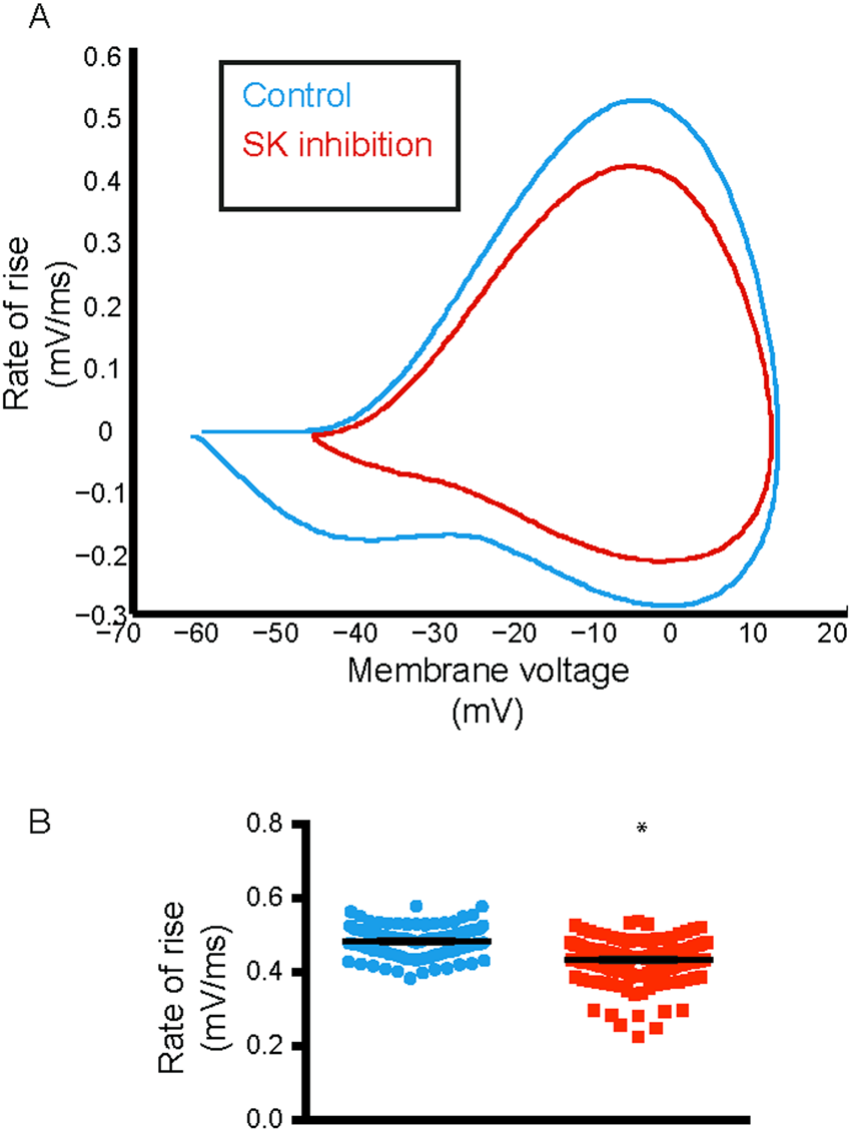
SK channel inhibition causes a decrease in rate of rise in membrane voltage. Phase-plane plot showed a clear reduction in rate of change in membrane voltage during a simulated SK channel inhibition (*red*) compared to control (*black*). (**B**) This was quantified by a significant decrease in the maximum value of rate of rise of membrane voltage, which is indicative of sodium channel unavailability. Error bars indicate mean ± S.E.M, not visible due to small errors.

A plot of membrane voltage against its rate of change (phase-plane plot) is another way of looking at membrane voltage dynamics that can shed light on channel availability and action potential threshold, which may not be easily deciphered from a spike train. We therefore wanted to compare the average phase-plane plot obtained from spontaneous firing with that produced after SK channel inhibition (Fig. 5A). Indeed the rate of rise in membrane voltage was significantly reduced (Fig. 5B) with a simulated block of SK channel (maximum rate of rise: without SK channel inhibition = 0.49 ± 0.003 mV/ms; with SK channel inhibition = 0.43 ± 0.004 mV/ms, paired student’s t-test, p < 0.05), which is a strong indication of reduced availability of sodium channels. This also suggested that if indeed there were reduced sodium channels available during spiking, action potential threshold must also be depolarised. We therefore next analysed action potential threshold.

**Figure 6:**
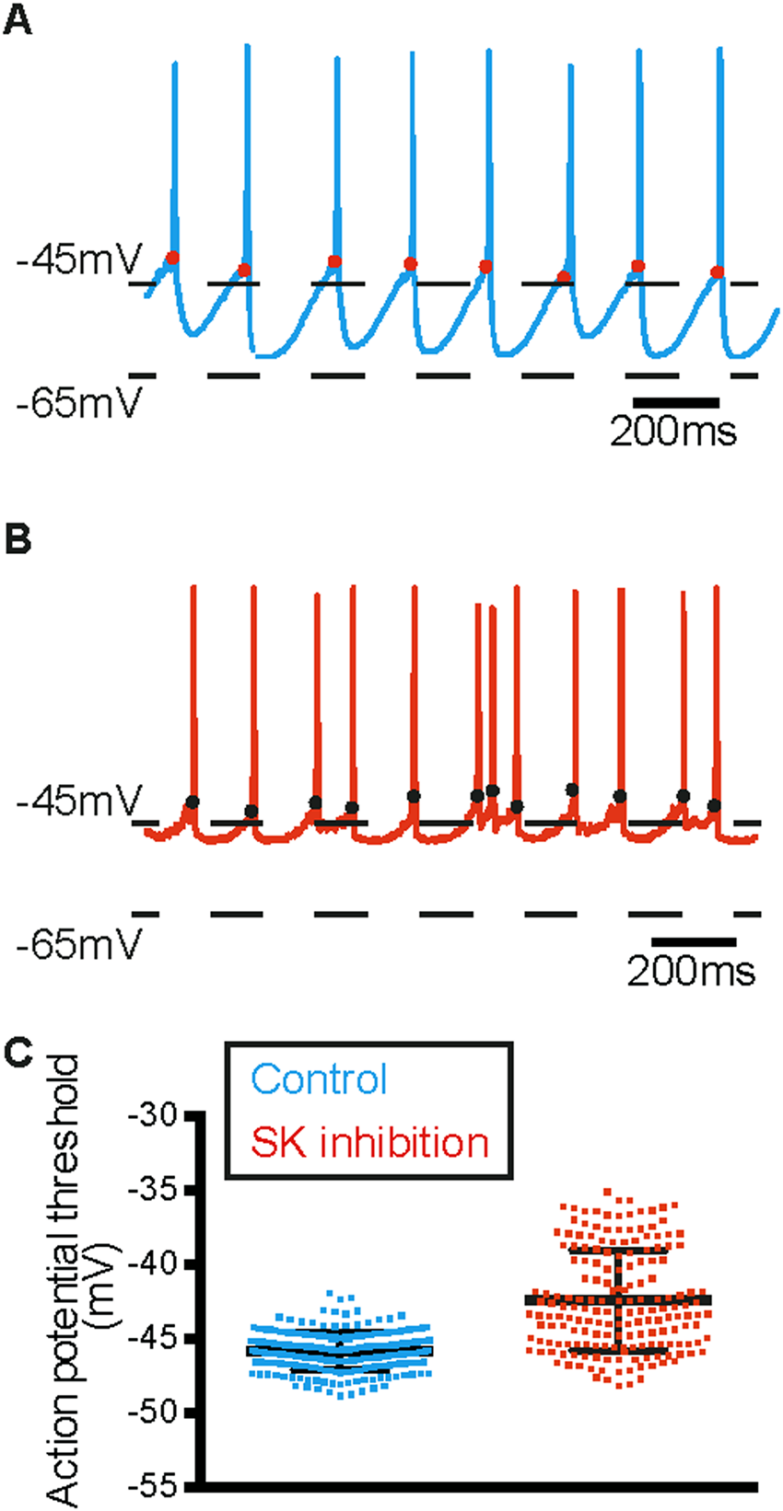
Action potential threshold depolarises and becomes more variable with simulated SK channel inhibition. (**A**,**B**) When SK channels were inhibited in the model, action potential threshold depolarised and became more variable (**B**) compared to control (**A**). The red and black dots indicate spike threshold. (**C**) Quantification of depolarisation in spike threshold as an effect of SK channel block in the model. Error bars represent mean ± S.E.M, not visible due to small errors.

A depolarisation in the average threshold for action potential generation was also observed when SK channel is blocked in our model (Fig. 6A,B). The mean action potential threshold depolarised from −45.06 ± 0.08 mV to −43.83 ± 0.08 mV (p < 0.05, paired student’s t-test). A visual inspection of the thresholds as plotted on individual spikes (Fig. 6B) showed spike-to-spike variability and so we wanted to quantify this. The CV of spike threshold also increased from 0.06 to 1.8. The effect of apamin on action potential threshold has not been reported in experiments in midbrain dopamine neurons and so this effect is also an important prediction of our model.

### SK channel inhibition causes insufficient recovery from inactivation of sodium channels

**Figure 7:**
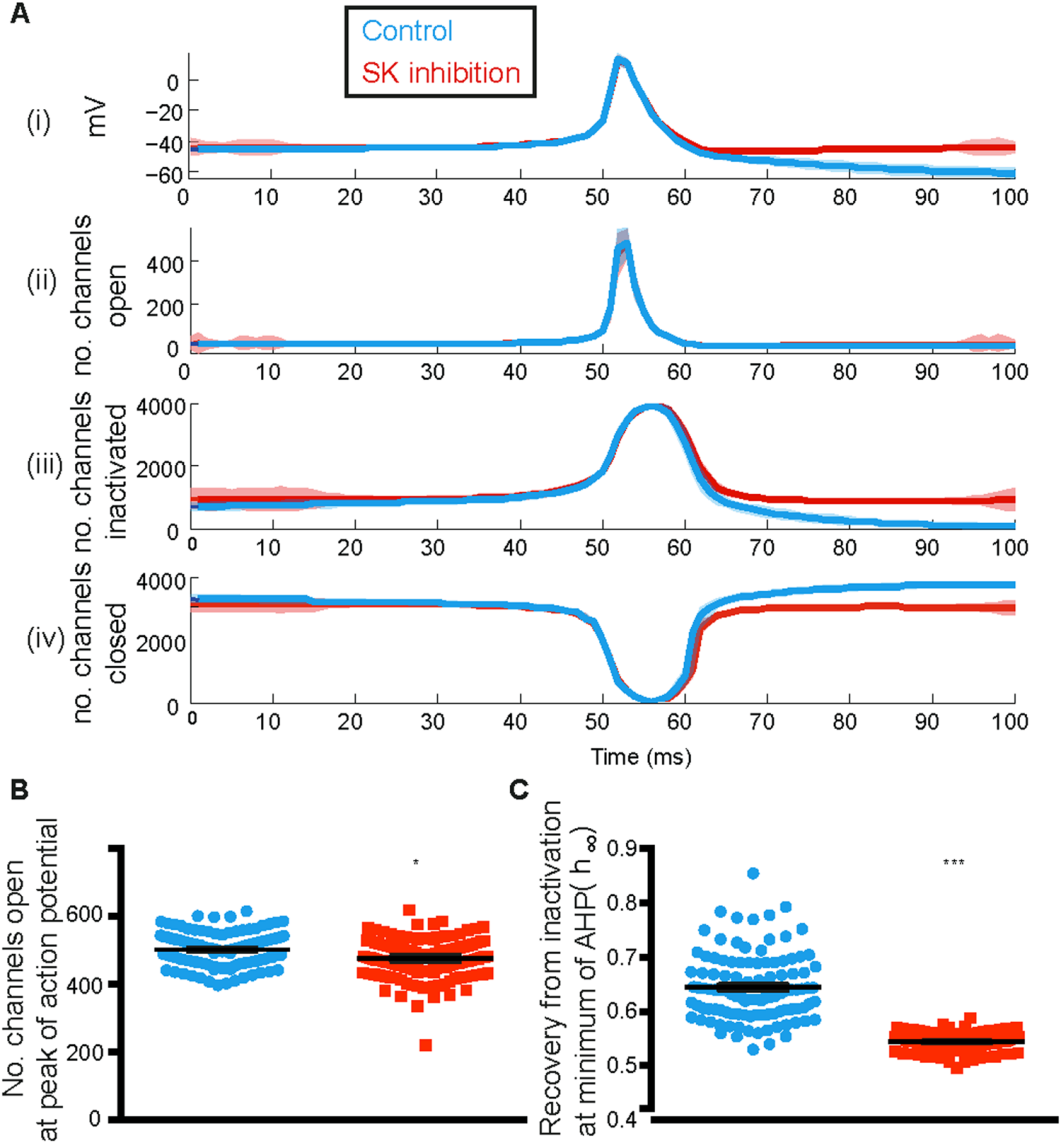
Failure of recovery from inactivation of sodium channels underlies decrease in firing regularity with SK channel inhibition. Shows the availability of sodium channels (*ii*–*iv*) during the course of the action potential (*i*). The number of sodium channels open increased steadily with depolarisation (*ii*) following the start of the action potential. This was accompanied by a steady increase in the number of inactivated channels (*iii*). While the number of open channels went back to zero as soon as the action potential had passed, there was a significant difference between the number of channels that remained in the inactivated state (*iii*). As the channels recovered from inactivation, they became *closed* (*iv*); however once again, a large number of channels failed to go into the *closed* state because they remained *inactivated*. Shaded region in (**A**) represents standard deviation around the mean. (**B**) Although we cannot see a reduction in the number of *open* channels during the peak of the action potential, there is a small yet statistically significant reduction in the number of *open* channels during the spike. (**C**) The reduction in recovery from inactivation is explained by the reduction in the *h* parameter of sodium channels inactivation dynamics. *h* was calculated at minimum membrane voltage attained after spike. Error bars represent mean ± S.E.M, not visible due to small errors.

Now that we had an indication of reduced sodium channel availability, we were able to investigate the reduction in sodium channel availability in more detail, given the underlying stochastic Markov model for the sodium channel. We therefore looked at the number of sodium channels in each of the open, closed, and inactivated states during the action potential (Fig. 7A(i)). We found that as the membrane remained hyperpolarised before the spike, a large number of sodium channels remained *closed*; next as the membrane began to depolarise, a number of sodium channels began to *open* to fire the spike (Fig. 7A(*ii*)). Once the spike started to repolarise, the number of *open* channels returned to minimum, thereby increasing the number of channels moving into the *inactivated* state (*iii*). However, when SK channels were inhibited, the number of channels in the *closed* state was reduced (*iv*) and more variable in the AHP region as shown by the shaded region due to a large number of them failing to recover from *inactivation* (*iii*). Moreover, there was increased variability in the number of channels *open* during the post-spike AHP, during SK channel inhibition, indicating that sometimes there are enough sodium channels to open in order to fire a spike while sometimes there are not (Fig. 7A (*ii*), *red*). This was primarily because those sodium channels did not go back to the *closed* state, and failed to recover from *inactivation*, due to the decreased AHP portion of the spike (Fig. 7A (*iii, iv*)). This meant that there were fewer channels available to *open* during the subsequent spikes.

We also found that the total number of open channels during the spike was reduced, and while the amount of reduction is only small, it was still statistically significant (Fig. 7B). Moreover, the variability in the number of ion channels in the *open* state is of greater importance because this shows that sometimes there are enough channels *open* to fire a spike while sometimes this is not the case. This is what is instrumental in giving rise to the irregularity associated with the spike train during SK channel inhibition. The inactivation variable h in sodium channel dynamics indicates amount of recovery from inactivation – a value of 1 indicating complete recovery. We therefore also looked at the amount of recovery from inactivation for both cases with and without SK channel inhibition at the point of peak AHP. We can see from Fig. 7C that during SK channel inhibition, recovery from inactivation at peak AHP was significantly reduced compared to control.

### SK channel inhibition causes reduction in A-type sub-threshold potassium current

A-type potassium channels activate at sub-threshold membrane voltages after they have recovered from inactivation during the AHP. Insufficient AHP due to SK channel inhibition must result in a reduced availability of A-type potassium channels, thereby promoting further increase in firing rate observed with SK channel block. In our model, SK channel inhibition resulted in a significant decrease in A-type potassium channel current (average peak current: without SK channel inhibition = 0.035 ± 0.0004 nA; with SK channel inhibition = 0.031 ± 0.0004 nA; p < 0.05, paired student’s t-test) that reflected a lack of A-type potassium channel activation. This was an important prediction of the model. Decrease in firing regularity is accompanied by increased firing rate in many experiments with apamin-induced block of SK channels in midbrain dopamine neurons, although this has not been related to decreased A-type potassium channel activity. However, reduced availability of A-type potassium channels during SK channel inhibition was demonstrated and related to the increase in firing rate observed with a simulated SK channel inhibition in the globus pallidus neuron model^28^.

## Discussion

Our simulation results suggest that the stochastic dopamine neuron model produces electrophysiological features commonly associated with midbrain dopamine neurons and responds well to simulated pharmacological manipulations thereby reinforcing that the model captures underlying ion channel dynamics in accordance with those reported in experiments. The spontaneous firing properties, such as the firing rate and more importantly the firing regularity, associated with the model dopamine neuron, agree well with experimental values suggesting that adding stochasticity to sodium and delayed rectifier potassium channels alone is sufficient to capture the physiological variability present in these neurons.

While majority of the results from the simulated pharmacological manipulations agreed well with experimental results and reinforced that the ion channels driving the model were calibrated well, inhibition of the L-type calcium channel conductance produced decreased firing regularity in the model that has not been reported often in experiments, except see ref. 69. While L-type calcium channels provide the bulk of intracellular calcium in dopamine neurons^79^, and are associated with SK channel activation^69^, many other types of calcium channels also serve as a source of intracellular calcium^79, 80^ that is used in turn by calcium-dependent SK channels for their activation. Therefore when one type of calcium channel is inhibited there may be other calcium channels that compensate for the loss in intracellular calcium and therefore SK channels would still be activated to provide the hyperpolarisation after a spike to regulate firing precision. For example, T-type calcium channels have also been shown to be functionally coupled to SK channels^80^. Because in the model used in this study, only the L-type calcium channel acts as the primary source of intracellular calcium that activates the SK channel, it is intuitive to see a decrease in firing regularity with its inhibition.

Having calibrated and tested the model, we next wanted to test and analyse whether and more importantly how SK channel inhibition results in a decrease in firing regularity in midbrain dopamine neurons. The firing pattern of a neuron is both a result of its intrinsic membrane properties as well as the sum of its synaptic inputs from other neurons^81, 82^. Most midbrain dopamine neurons fire spontaneously in the presence and absence of extrinsic synaptic inputs^53, 83–85^ and dynamically modulate their firing activity by changing their firing rate and pattern in response to stimuli such as rewards^8^ and aversive events^10^. While many experimental studies have demonstrated that SK channels appear to selectively control firing regularity in midbrain dopamine neurons, no hypotheses have been put forth as to *why* or *how* this effect is mediated by SK channels. It could be argued that during inhibition of SK channels, membrane voltage values would lie mostly in the steep region of a sodium channel gating curve for a large portion of the ISI and therefore stochastic phenomena in this region can result in irregular spiking. Whereas, in the presence of a large AHP as a result of SK channel current, membrane voltage is in the flat regions of this curve where stochastic phenomena are observed, thus maintaining regular spiking.

Sodium ions are essential for spike generation in midbrain dopamine neurons^86^. As the membrane is depolarised, increasing numbers of sodium channels are activated until enough are available or *open* to fire an action potential in an all-or-none manner; once the action potential peaks, sodium channels begin to *inactivate* ^87, 88^. Once *inactivated*, the neuronal membrane needs to be hyperpolarised to facilitate recovery of these channels from *inactivation* in order to be available for *activation* when the next round of depolarisation occurs^88^. Potassium currents flowing through SK channels provide the bulk of the post-spike hyperpolarising drive that helps in recovery from *inactivation*.

We therefore hypothesised that the AHP mediated by SK channel current provides enough hyperpolarisation to help sodium channels recover from inactivation after the preceding spike such that there are always enough available to *activate/open* to fire subsequent spikes at regular intervals. Our stochastic model not only produces a spike train with variability in ISI as seen in biological dopamine neurons but also demonstrates the changes in regularity of firing when we simulate an SK channel inhibition. The reduction in AHP gives rise to irregularity in firing, and also increases the number of action potentials fired, a feature that is commonly observed in experiments, but not always. At the molecular level, it has been demonstrated that two different subtypes of SK channels also present in dopamine neurons – SK2 and SK3 - differentially control the firing rate (SK3) and precision (SK2 and SK3)^77^. The inconsistent effect of SK channel block on firing rate observed in experiments could depend on the relative and/or co-expression of these two SK channel subtypes in the neurons recorded from.

In our model dopamine neuron, an increase in firing rate is expected because of a reduction in the time to reach action potential threshold as a result of a reduced AHP. In addition, the *relative* availability of sub-threshold ion channels is also critical in determining the effect on firing rate after SK channel inhibition as demonstrated in their computational study^28^ in globus pallidus neurons where the proportion of open *sub-threshold* sodium and potassium channels determines the firing rate after a simulated SK channel inhibition. Similarly, our simulations also demonstrate a reduction in sub-threshold activated A-type potassium channel, which explains the increase in firing rate, which is an important prediction of our model. While SK channel inhibition resulted in a reduction in A-type potassium current thereby perhaps resulting in an increase in the firing rate of the model neuron, modulation of the A-type potassium conductance can have other important consequences *in vivo*, such as increased excitability in turn making the neuron more susceptible to burst firing. This could support the fact that pharmacological inhibition of SK channels alone sometimes also facilitates burst firing^47, 76^.

Another interesting result from our modelling study is that of SK channel’s role in regulating and maintaining action potential threshold. SK channels not only maintain variability in firing, but also seem to minimise variability in spike threshold. This however seems intuitive because if SK channels control availability of spike producing ion channels such as the sodium channels, it is expected to have an effect on spike threshold as sodium channel inactivation alone has been shown to cause variability in spike threshold^89^. Moreover, the depolarisation in threshold due to slowing of membrane dynamics after SK channel block also agrees well with the fact that membrane voltage and spike threshold have an inverse correlation^90^. This is referred to as threshold adaptation and has profound effects on the neurons input-output properties such as enhanced coincidence detection^91^, improved feature selection^92^, and temporal coding^93^. Therefore our results suggest that SK channels regulate and reduce the variability of spike threshold in midbrain dopamine neurons.

Our results have demonstrated how a single ion channel exerts its influence on other ion channels and regulates the firing properties of the neuron. Our results also highlight the importance of ion channel noise in dopamine neuron electrophysiology indicating that channel noise is sufficient to explain *how* SK channels modulate the neurons’ firing regularity. The increased variability that arises in a dopamine neuron’s firing code due to SK channel inhibition could have potential consequences at the behavioural level, for example, affecting the trade-off between exploration and exploitation, especially due to increased burst firing that sometimes accompanies decreased firing regularity^23, 24, 94–96^.

## Methods

While it is important and might seem practical to take into consideration the full morphology of a neuron to precisely reproduce its physiology *in silico*, modelling only the soma can be sufficient, as a first step, in cases like ours where there is enough evidence to suggest that the soma contains all the ion channels necessary to produce the electrophysiological features of interest^31, 41, 63, 66^. Because we were investigating ionic conductances underlying regulation of firing precision in midbrain dopamine neurons, we have used the Hodgkin-Huxley (HH) formalism to simulate ion channel dynamics. In particular, our hypothesis required that we model conductances as individual ion channels, especially those that could be modulated by the SK-mediated AHP such as the sodium, as independent stochastic entities capable of existing in many voltage-dependent states like *open*, *inactivated*, and *closed*, instead of simulating the average gating behaviour of each conductance as is usually seen in deterministic neuronal models^31, 33–35, 66^. In this study, we chose to model the spike generating sodium and potassium channels as stochastic to test the hypothesis that it is the availability of these spike generating channels that is most affected when the AHP is inhibited by an SK channel block.

We therefore developed a hybrid biophysical dopamine neuron model driven by a combination of stochastic and deterministic ion channels. A single spherical compartment of diameter 1 μm and capacitance 1 μF/cm^2^ represents the dopamine neuron soma and holds some of the ionic conductances known to be present in midbrain dopamine neurons (Fig. 1A) and more importantly those that are sufficient to reproduce the basic electrophysiological features associated with these neurons. The equivalent electrical circuit is illustrated in Fig. 1B. The membrane is considered as an iso-potential patch with ion channels distributed uniformly across the membrane. While most of the ion channels in the model are *deterministic* (i.e., their gating describes the average behaviour of a population of ion channels as a single lumped conductance), each individual fast sodium and delayed rectifier potassium channel in our model has been described as a *stochastic* Markov process^97^, that can be in one of many states such as *open*, *closed*, *inactivated*, the transitions between which are determined by associated voltage-dependent probability rate functions^98^. Intracellular calcium dynamics are modelled as a single pool of calcium ions coming into the neuron through the opening of voltage-gated L-type calcium channels. Calcium ions are subsequently removed using a decay term that lumps together more complex phenomena like extrusion by pumps and intracellular buffering^99, 100^. The activation of SK channel is modelled by assuming that calcium binds to the SK channel with Michaelis-Menton type kinetics with a Hill coefficient of 4^101^. The model was implemented in the C++ programming language using our own stochastic simulator Modigliani (http://www.doc.ic.ac.uk/~afaisal/FaisalLab/Modigliani/modigliani-api/index.html).

### Modelling stochastic ion channels

Stochastic ion channel gating can be modelled as a Markov chain that describes a channel as finite state stochastic process^29, 102^, giving rise to Markovian gating schemes like the 8-state scheme for a sodium channel and 5-state scheme for a potassium channel (Fig. 1C,D). In such Markovian schemes, the individual states can be interpreted as local energy minima of the channel conformation and the transitions between states can be related to the crossings between energy barriers^103^. Biophysically, every state corresponds to a specific bound/unbound state of the ion channel, and each transition is the binding or unbinding of one gating particle. Markov models can be recovered from deterministic gating-particle models as described in ref. 29. Briefly, deterministic models are reformulated as a specific subclass of Markov models^104, 105^ as described here. Every possible combination of bound and unbound gating particle corresponds to a discreet channel state and kinetic rate functions that describe the binding and unbinding rate of gating particles are correspondingly used to describe the probability of transition between ion channel states. Because gating particle models assume that individual particles of the same type are indistinguishable and independent of each other^87^, multiple states can therefore have the same number of bound particles of each type and so such states can be lumped into a single Markov state^29^. To account for the lumped states, transition rates are multiplied by a factor *k*, which is determined as follows. A transition corresponding to the unbinding of a gating particle of type *j* has a factor *k* that equals the number of particles of type *j* bound to the state where the transition originated. Conversely, the transition corresponding to the binding of a gating particle of type *j* has a *k* that equals the multiplicity *l*(*j*) minus the j particles bound at the target state. This procedure allows for the transformation of any deterministic model into stochastic model of ion channel gating.

The stochastic channel gating is implemented using the Binomial algorithm^29^. The binomial algorithm assumes that ion channels of the same type belong to an independently and identically distributed population and a transition from one state to another is calculated over the entire channel population instead of each channel at a given time. This means that a binomially distributed number of channels switch state at each iteration of the simulation^44, 45^. In other words, a single binomially distributed random variable can determine the number of channels that switch states at each time step^29^. An integration time step of 0.001 ms was used in our simulations. This value of time step size gives a good approximation to experimental data without being computationally too expensive. Simulations with other step sizes are also shown in Supplementary Fig. S1.

### Model parameters and rate functions

The model parameters were initially adapted from existing computational models of dopamine neurons^31, 33, 66^ and subsequently modified to fit our model’s dynamics. The model neuron contains 6 ion channels namely the fast activating and inactivating sodium channel and the non-inactivating delayed rectifier potassium channel, both of which are modelled as a population of stochastic entities capable of existing in many states, the A-type potassium channel, the L-type calcium channel, the SK channel and the leak. The maximal conductances and reversal potentials for the above currents are listed in Table 1.

**Table 1:** Maximal conductances and reversal potentials used in the model.

	Na	K_dr_	K_A_	CaL	SK	leak
$\bar{g}$(mS/cm^2^)	—	—	4	5	5	0.3
E (mV)	55	−72	−75	50	−75	−45

The fast sodium current plays a major role in action potential initiation and propagation^106^ and the delayed rectifier potassium channel current actively repolarises the action potential^63, 106^ in midbrain dopamine neurons. Because our hypothesis states that the AHP provided by the SK current regulates the availability of other voltage gated ion channels that are involved in spiking, we modelled the sodium and potassium currents as stochastic entities. This enabled us to analyse how the availability of these channels changes in the absence of the post-spike AHP during SK channel inhibition. Single channel conductances and channel densities for the stochastic currents are listed in Table 2.

**Table 2:** Single channel conductances and densities for the stochastic currents.

	Na	*K*_*dr*_
Single channel conductance (pS)	12	2
Density (cm^−2^)	12	6

The current flowing through ion channels is generally computed using Ohm’s Law such that

1 $${I}_{ion}={g}_{ion}({V}_{m}-{E}_{ion})$$

where g_*ion*_ is the instantaneous ion channel conductance, V_m_ is the membrane voltage and E_*ion*_ is the reversal potential for that ion. In the case of *stochastic* ion channel gating, g_*ion*_ is the product of maximal ionic membrane conductance $\bar{g}{}_{ion}$ and p_*ion*i_(*V*_*m*_), the voltage-dependent probability of a single channel being open,

2 $${g}_{ion}={\bar{g}}_{ion}{p}_{ioni}({V}_{m})$$

where $\bar{g}\,$_*ion*_ is the product of single channel conductance γ_i_ and ρ_i_, the number of channels in unit membrane area or channel density. Therefore we can write

3 $${g}_{ion}={\gamma }_{i}{\rho }_{i}{p}_{ioni}({V}_{m})$$

The probability p_*ion*i_(*V*_*m*_) that an ion channel is open is a product of the individual probabilities that each gate of the channel is in the bound or activated state in a Markovian gating scheme because each gate is considered independent of each other. If q_j_ is the probability that a single gating particle j is in the bound state then,

4 $${p}_{ioni}({V}_{m})={q}_{j}{({V}_{m})}^{l}$$

where l is the number of gates of type j. q_j_ in turn is governed by the linear differential equation of the form:

5 $${\dot{q}}_{j}={\alpha }_{j}({V}_{m})(1-{q}_{j})-{\beta }_{j}({V}_{m}){q}_{j}$$

where α and β are rate functions that describe the rate of change of q_j_ measured in the order of msec^−1^.

Given below are the descriptions for all the currents used in our model.

*Sodium channel - I*_*Na*_

6 $${\alpha }_{m}=-0.1({V}_{m}+29.7)/(\exp (-({V}_{m}+29.7)/10)-1)$$

7 $${\alpha }_{h}=0.07(\exp (-({V}_{m}+48)/20))$$

8 $${\beta }_{m}=4\exp (-({V}_{m}+54.7)/18)$$

9 $${\beta }_{h}=1/(1+\exp (-({V}_{m}+18)/10))$$

*Delayed rectifier potassium channel - I*_*Kdr*_

10 $${\alpha }_{n}=-0.01({V}_{m}+45.7)/(\exp (-({V}_{m}+45.7)/10)-1)$$

11 $${\beta }_{n}=0.125(exp(-({V}_{m}+54.7)/80))$$

*A-type potassium current - I*_*A*_

12 $${I}_{A}={\bar{g}}_{A}{a}^{4}b({V}_{m}-{E}_{A})$$

13 $${a}_{\infty }=1/(1+\exp ((60-{V}_{m}-42)/15));{\tau }_{a}=10$$

14 $${b}_{\infty }=1/(1+\exp (-({V}_{m}+43)/20))$$

15 $${\tau }_{b}=2\,exp(-{({V}_{m}+50)}^{2}/50)+1.1$$

*L-type calcium current - I*_*CaL*_

16 $${I}_{CaL}={\bar{g}}_{CaL}{a}_{CaL}({V}_{m}-{E}_{CaL})$$

17 $${a}_{\infty }=1/(1+\exp ((-{V}_{m}+55)/5))$$

18 $${\tau }_{a}=18\exp (-{({V}_{m}+45)}^{2}/625)+1.5$$

*Calcium-activated potassium current - I*_*SK*_

19 $${I}_{SK}={\bar{g}}_{SK}{a}_{SK}({V}_{m}-{E}_{SK})$$

20 $${a}_{SK}=\frac{{[Ca]}_{i}^{4}}{{[Ca]}_{i}^{4}}+{0.2}^{4}$$

where [Ca]_i_ is intracellular calcium concentration, governed by the equation

21 $$d/dt{[Ca]}_{i}=(-{I}_{CaL}/2Fv)-\beta {[Ca]}_{i}$$

where F is Faraday constant, v is volume of the soma and β□□□ the time constant of [Ca]_i_ extrusion.

*Background leak - I*_*L*_

22 $${I}_{L}={\bar{g}}_{L}({V}_{m}-{E}_{L})$$

We calibrated our model by simulating current approximations to voltage-clamp data obtained from published dopamine neuron literature^63, 79, 107^ for all of the ion channels included in our model. When recording simulated currents from an individual ion channel type, the maximum conductance of all the remaining channels was set to 0, including leak, so that the current responses represented leak-subtracted values as is normally done in experiments. Voltage clamp protocols were adapted from existing published studies characterising ion channels expressed in midbrain dopamine neurons either in SNc or VTA.

### Data analysis

**Figure 8:**
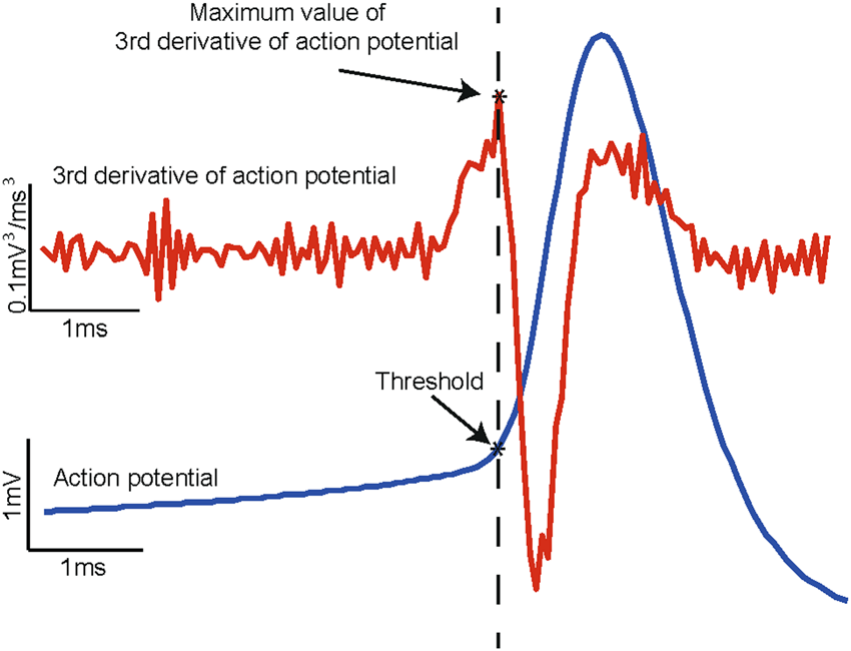
Method for calculation of action potential threshold. Action potential is calculated as the voltage corresponding to the maximum value of the third derivative of the membrane potential.

Customised software was written in MATLAB **(**MATLAB and Statistics Toolbox Release 2012b, The MathWorks, Inc., Natick, Massachusetts, United States) for all spike train and action potential related analyses. Spike train histograms and CV of ISI were computed in the presence of SK channel current, ($\bar{g}$ = 5 mS/cm^2^) and with SK channel inhibition ($\bar{g}$ = 0.5 mS/cm^2^) in order to study the effects of a simulated block of SK channels. CV of ISI has historically been used as a measure of firing regularity^108^ and is calculated as the standard deviation (SD) of ISI divided by the mean of ISI. Action potential threshold was measured as the voltage at which the third temporal derivative of membrane voltage reaches maximum^109^ (Fig. 8). This method of threshold calculation is more robust and precise compared to choosing a safe, yet arbitrary, value of membrane voltage that appears to be the threshold upon close visual inspection of spikes and/or phase-plane plots^109^. All statistical analyses were performed on a spike train consisting of 100 consecutive spikes unless mentioned otherwise. Statistics was performed in GraphPad PRISM version 6, GraphPad Software, San Diego California USA, www.graphpad.com. Group means are presented as mean ± SEM and two group comparisons were performed using paired student’s t-test with probability level of *p* < 0.05 qualifying as statistically significant.

## Electronic supplementary material

Supplementary information
