# Supplementary material for: Calcium-activated SK channels control firing regularity by modulating sodium channel availability in midbrain dopamine neurons
Source: Sci Rep. 2017 Jul 12;7:5248. doi: 10.1038/s41598-017-05578-5 (PMC5507868; doi:10.1038/s41598-017-05578-5)
Supplement: Supplementary file 1 — Supplementary information [file 41598_2017_5578_MOESM1_ESM.doc]

**Calcium-activated SK channels control firing regularity by modulating sodium channel availability in midbrain dopamine neurons**

Rajeshwari Iyer (RI)^1,2^, Mark A Ungless (MAU)^1,2, *^, Aldo A Faisal (AAF)^1, 2, 3,4 *^

^1^ MRC London Institute of Medical Sciences (LMS), Hammersmith Hospital Campus, Du Cane Road, London W12 0NN, UK

^2^ Institute of Clinical Sciences (ICS), Faculty of Medicine, Imperial College London, Du Cane Road, London W12 0NN, UK

^3^ Department of Bioengineering, Imperial College London, London, United Kingdom

^4^ Department of Computing, Imperial College London, London, United Kingdom

^*^ Joint senior authors

**Corresponding authors**:

Mark A Ungless: [mark.ungless@imperial.ac.uk](mailto:mark.ungless@imperial.ac.uk)

Aldo A Faisal: [a.faisal@imperial.ac.uk](mailto:a.faisal@imperial.ac.uk)

**Supplementary Fig S1:**
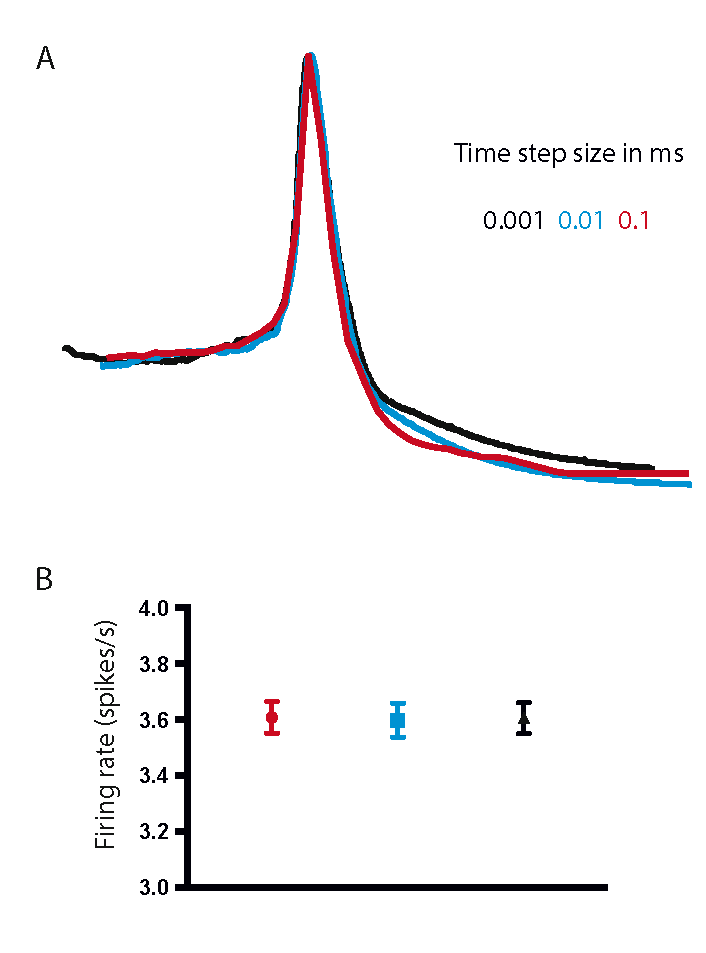
**Action potential shape and firing rate are not significantly different at different time step sizes**

(A) Average action potentials obtained from simulations at three step sizes 0.1ms (red), 0.01ms (cyan), and 0.001ms (black) aligned at the point of maximum rate of rise do not show much variability depending on the step size of the simulation. (B) There is no statistical difference in the average firing rate obtained from multiple trials of the simulations with different time step size as above. Number of simulations = 30 at each time step size.
